# The recurrence of well-differentiated liposarcoma from benign giant intramuscular lipoma: A case (CARE-compliant) report

**DOI:** 10.1097/MD.0000000000024711

**Published:** 2021-02-12

**Authors:** Yeon Ji Lee, Won Jin Cha, Yesol Kim, Deuk Young Oh

**Affiliations:** a Department of Plastic and Reconstructive Surgery, St. Vincent hospital, College of Medicine, The Catholic University of Korea; b Department of Plastic and Reconstructive Surgery, Uijeongbu St. Mary's Hospital, College of Medicine, The Catholic University of Korea; c Department of Plastic and Reconstructive Surgery, Seoul St. Mary's Hospital, College of Medicine, The Catholic University of Korea, Seoul, Republic of Korea.

**Keywords:** giant lipoma, intramuscular lipoma, malignant transformation, pedicled latissimus dorsi muscle flap, recurrent liposarcoma, WDLS, well-differentiated liposarcoma

## Abstract

**Rationale:**

Recurrent liposarcoma, previously confirmed as lipoma, has rarely been reported. However, the risk factors for recurrence and the correlation between benign lipoma and malignant liposarcoma remain unclear. In this case study, we suggest a precise diagnostic strategy to minimize recurrence and malignant transformation.

**Patient concerns:**

A 60-year-old male patient with a history of left chest wall swelling without any symptoms underwent excisional surgery, and the mass was confirmed as a benign lipoma in 2015. In 2019, the patient returned to the hospital with symptoms of a palpable mass on the left chest wall.

**Diagnosis:**

The mass was considered a recurrent lipomatous tumor with the possibility of malignant transformation. Magnetic resonance imaging (MRI) revealed a deep-seated, septate, intramuscular, irregular margin, and large lipomatous tumor invading the ribs, pleura, and adjacent muscle, suggestive of malignancy. The MRI findings were similar to those 4 years ago, except for margin irregularity and invasion to adjacent tissue.

**Interventions:**

Wide en bloc excisions encompassing the 5th to 7th ribs, pleura, and adjacent muscle were followed by reconstruction with a pedicled latissimus dorsi muscle flap.

**Outcomes:**

The recurrent large lipomatous tumor was confirmed as well-differentiated liposarcomas through histological and MDM2-FISH immunohistochemical staining. Postoperatively, follow-up visits continued for 1.5 years without recurrence.

**Lessons:**

We suggest that deep-seated, septate, and giant lipomatous tumors should be considered as risk factors for recurrence with the possibility of malignancy and misdiagnosis. It is important to inform patients of all these possibilities and plan close and long-term follow-up.

## 1 Introduction

Benign lipoma is one of the most common mesenchymal neoplasms, with a prevalence rate of 1%, involving mostly patients in their 40 second and 60 second, with varying sizes and locations and often without symptoms.^[1]^ Although most of them are located in supercritical subcutaneous layers, it is not easy to distinguish large and deep-seated intramuscular lipomas from well-differentiated liposarcomas (WDLSs, atypical lipomas) before surgery.

Based on the World Health Organization (WHO) classification of adipocytic tumors, WDLS is considered an intermediate malignant soft tissue neoplasm. It is the most common sarcoma accounting for 10% to 15% of all cases ^[2,3]^ and is usually associated with locally aggressive recurrence.^[4]^

The differential diagnosis of benign lipoma and WDLS is difficult based on preoperative imaging studies alone, especially if they are large in size, located in the intramuscular layer, or are septate with unclear boundaries.^[5]^ Moreover, it could be difficult to distinguish these 2 masses completely via intraoperative frozen section examination because of similar histological characteristics.^[6]^ Histologically, WDLS is characterized by mature fat with a variable number of spindled cells displaying hyperchromatic nuclei and multi-vacuolated lipoblasts, testing positive for CDK4 (average 62.1%) immunohistochemically.^[7]^ Most benign lipomas consist of mature adipocytes, but a few lipomas are similar to WDLS not only in histology but also in immunochemistry.^[6]^ making it difficult for many surgeons to distinguish between benign and malignant cases.

Recurrence of lipomatous tumor as malignant liposarcoma after surgical excision of benign lipoma has rarely been reported.^[8]^ The incidence of recurrence is thought to be reduced by performing precise assessment considering the possibility of malignancy with the risk factors and understanding the correlation between benign lipoma and WDLS.

In an effort to understand the pathogenetic correlation between 2 masses, several recent studies have raised the possibility of a biological continuum of benign, atypical, and malignant mesenchymal neoplasms and malignant transformation from benign lipoma to WDLS.^[9]^ while other studies have reported the possible origin of these 2 masses from the same adipocytes with identical genomic background.^[6]^ On the other hand, there have been reports of co-existence of both masses.^[10–12]^ However, its pathogenesis remains unclear. In this case study, we present a case of recurrent liposarcoma that was surgically excised and confirmed as a benign lipoma. In this case, we want to discuss the correlation between benign lipoma and WDLS along with a differential diagnosis, to present the risk factors of recurrence, and to consider ways to reduce recurrence with malignant transformation.

## 2 Method

### 2.1 Ethical approval

This study was approved by the Institutional Review Board of the Catholic Medical Center Office of the Human Research Protection Program. (KC20ZASA0156) at the Catholic University of Korea (Seoul, Korea). All data were analyzed anonymously and according to the principles of the Declaration of Helsinki 1975 (revised in 2008).

The patient has provided informed consent for publication of the case.

## 3 Case report

**Figure 1:**
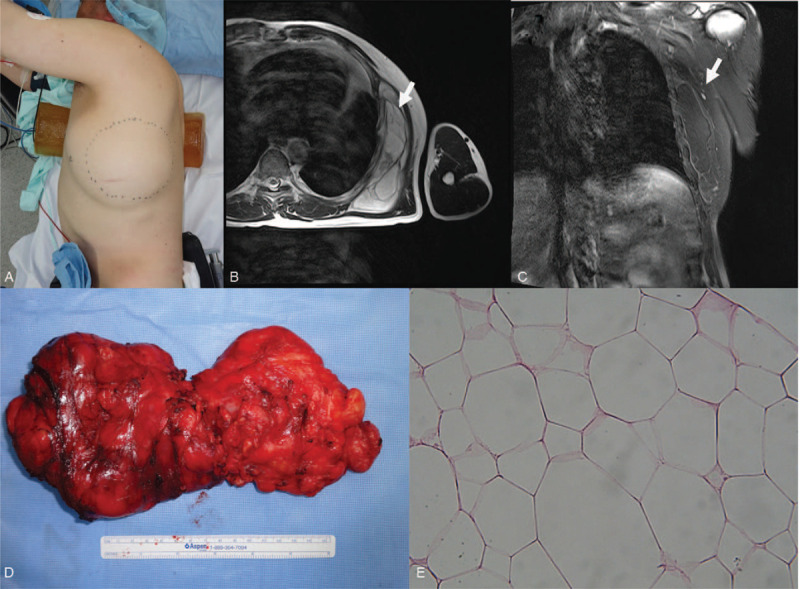
A 60-year-old male admitted with a swollen left chest wall without any symptom in 2015 (A). Based on the the magnetic resonance imaging (MRI) findings, an approximately 159 x 144 x 38 mm septate mass lying beneath serratus anterior muscle was identified in the left chest wall (B, C). A complete surgical excision was performed (D). The submitted specimen is an ovoid mass, measuring 160 x 85 x 75 mm with a pale-brown and a thin fibrotic capsule. In the pathological findings (x 400), the neoplasm was composed of mature white adipose tissue with few adipocytes suggesting a benign lipoma (E).

A 60-year-old man presented to the Department of Plastic and Reconstructive Surgery with a history of left chest wall swelling without any symptoms in 2015 (Fig. 1A). Based on the finding of magnetic resonance imaging (MRI), we found a septate mass measuring 159 x 144 x 38 mm lying beneath the serratus anterior muscle at the left chest wall (Fig. 1B, C). The mass was thought to be a benign lipoma on preoperative MRI, with low heterogeneity and regular margins. Surgical excision was performed under general endotracheal anesthesia (Fig. 1D).

After the operation, the excised tissue was diagnosed as benign lipoma, which was grossly defined by a pale brown and thin fibrotic capsule and a tan yellow fatty surface in the cross-section, suggesting a benign lipomatous neoplasm. Microscopically, the neoplasm was composed of mature white adipose tissue with few adipocytes (Fig. 1 E). The resected margins of the tumor were not evaluated as the lipomatous tumor was confirmed to be benign. After a month of postoperative follow-up, the patient did not visit our medical center for 4 years.

**Figure 2:**
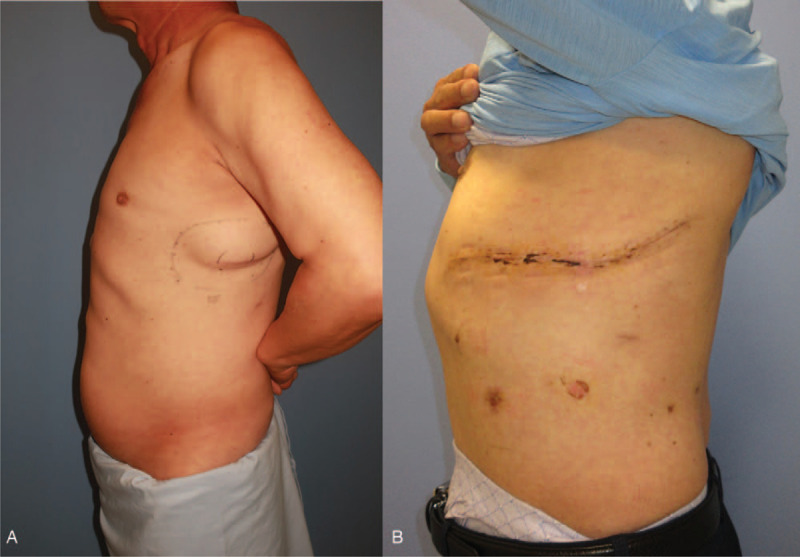
In 2019, the patient returned to hospital with a symptomatic mass on the left chest wall at the same site of prior excision (A). Wide en-bloc excision including pleura and 5th to 7th ribs and immediate reconstruction with pedicled latissimus dorsi muscle flap were performed. Postoperative photography is shown in (B).

**Figure 3:**
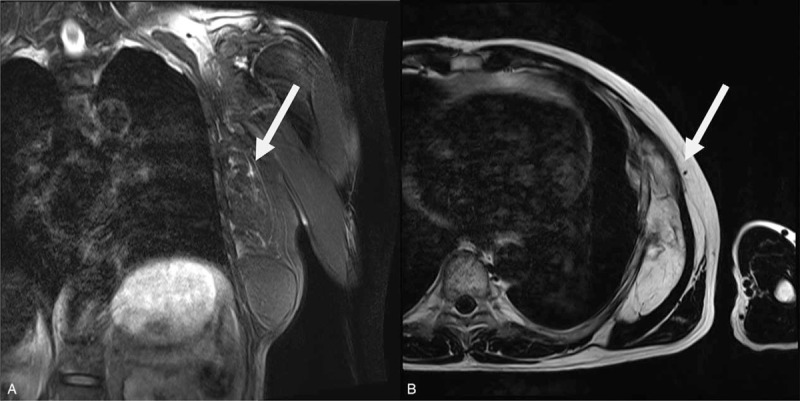
A septate mass measuring approximately 159 x 144 x 38 mm with a high SI on both T1- and T2WIs with fat-suppression in the left chest wall of MRI and protruding inside the thoracic cage, possibly involving the pleura (A: coronal section, B: axial section).

In 2019, the patient returned to the hospital with symptoms of globus on the left chest wall (Fig. 2A). Magnetic resonance imaging revealed septate masses measuring 160 × 114 × 66 mm in size and located in the left lateral chest wall at the site of prior excision, just beneath the serratus anterior muscles and protruding inside the thoracic cage, possibly involving the pleura. Marginal irregularities were detected. (Fig. 3A and 3B)

**Figure 4:**
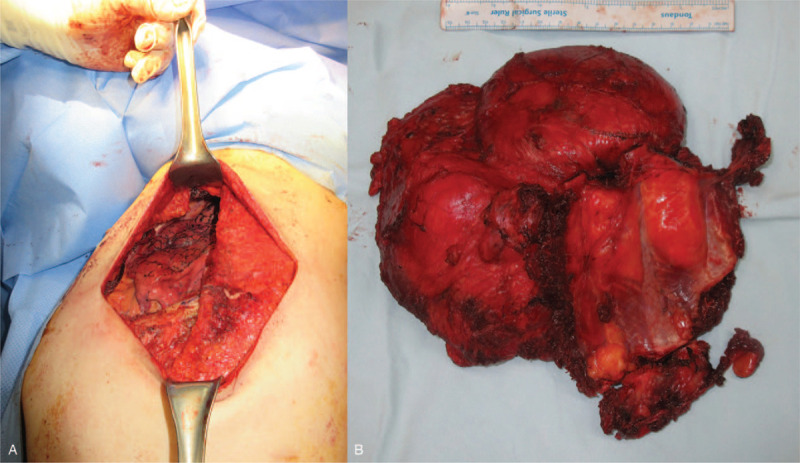
The large septate mass was located in the intramuscular layer with unclear border (A) Wide en-bloc excision was performed with resection of 5th-7th ribs, with the serratus lying anterior and adjacent to the pleural tissue (B).

Surgically wide excision containing resection of invaded ribs, pleural tissue, and muscles, and reconstruction of the chest wall was planned. We cooperated with the thoracic surgery team for pleural excision after confirming pleural invasion via microscopic frozen testing during the operation. The huge septate mass was located in the intramuscular layer with an unclear border (Fig. 4 A). We performed a frozen test, and a positive finding was reported on a resected pleural margin, suggesting malignancy. Wide en bloc excision was performed with a resection of the 5th to 7th ribs, and the serratus anterior and adjacent to the pleural tissue (Fig. 4 B). All resected margins of the tumor were confirmed to be negative for malignant cells. Pleural and rib defects were covered with a Marlex mesh (C.R. Bard, Inc., Covington, GA, USA). Skin and soft tissue defects were covered with a pedicled latissimus dorsi muscle flap.

**Figure 5:**
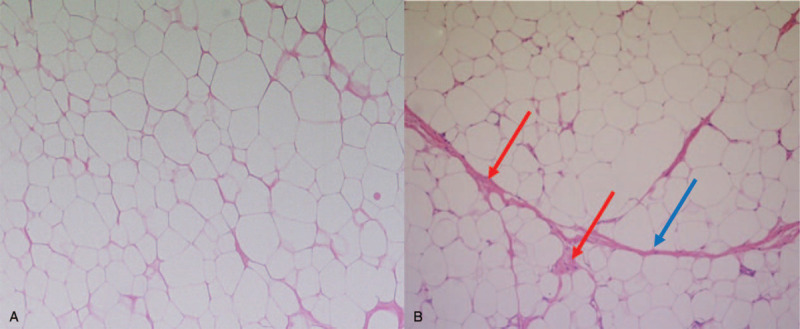
Pathologic finding (x 100) of excised tissue in 2015 shows the mature adipose tissue with few adipocytes suggesting a benign lipoma (A). Pathologic findings (x 100) of the excised tissue in 2019 show multiple septa (blue arrow) and variation in cell size, and focal adipocytic nuclear hyperchromasia (red arrow) suggesting WDLS (B).

Based on the final results, the mass was histologically diagnosed as WDLS with multiple septa (blue arrow) and variation in cell size, along with focal adipocytic nuclear hyperchromasia (Fig. 5 B). The histological difference between the benign lipomatous neoplasm excized 4 years before and the WDLS is shown (Fig. 5 A, B).

Immunochemical staining with MDM2-FISH was performed to examine gene amplification. MDM2 and chromosome 12 centromere (CEP12) using the Vysis LSI MDM2 Spectrum Orange Probe nd Vysis DNA FISH probe CEP12 (Abott Molecular/Vysis, Des Plaines, IL, USA) were examined, and.^[13]^ results of MDM2-FISH showed focally positive and 5.96 of MDM2/CEP12 ratio, suggesting that MDM2 gene amplification indicated the lipomatous tumor as WDLS.

The patient was discharged after postoperative care. Postoperatively, follow-up visits continued for 1.5 years. Follow-up CT for screening of recurrence was conducted a year postoperatively, and no evidence of recurrence was found. The patient remained stable without recurrence (Fig. 2 B).

## 4 Discussion

A case study of recurrent WDLS on the forearm flexor compartment was reported in which a benign lipoma was surgically excised 28 years ago. According to this study, the risk factor for recurrence with malignancies is presented as a large-sized (giant) lipoma of 5 cm or more, and unusual characteristics in the location of the tumor.^[8]^ In this study, a deep-seated, septate, and large-sized lipoma was detected on the MRI images in 2015. Although this tumor was considered to be a benign lipoma most likely due to low heterogeneity and regular margin findings, the features described above are thought to suggest a potential risk of recurrence. Therefore, considering these risk factors and performing precise preoperative evaluation is required to prevent the recurrence of lipoma with malignant transformation.

In the present case of deep-seated, septate, and large-sized lipomatous tumors, differential diagnosis from liposarcoma before surgery could be difficult with unclear radiological findings, and the possibility of liposarcoma cannot be completely excluded before surgery. Therefore, surgeons must select appropriate radiological examinations and understand the radiological findings suggesting malignancy. Among the radiological examinations, magnetic resonance imaging (MRI) has been considered the gold standard for the differential diagnosis of soft-tissue tumors.^[14]^ MRI images that suspect liposarcoma include size, depth, nodularity, or stranding as well as heterogeneity^[15]^; recently, based on radiomic-MR imaging, texture analysis could contribute to the advancement of differential diagnosis by quantifying the texture heterogeneity of lipomatous tumors.^[16,17]^

However, performing MRI on all patients visiting with soft-tissue tumors is less cost-effective, so it is necessary to select an appropriate screening modality according to the characteristics of the tumor. Based on this appropriate screening modality, a patient-specific approach is needed for accurate decision-making. According to the recently presented uncontrolled retrospective study, in which the differential diagnosis between WDLS and benign liposarcoma was performed using sonography imaging, sonographic findings were reported to predict WDLS with highly accurate correlation with pathological features. The features of malignancy are described as

1. deep location,
2. irregular shape,
3. hyperechogenicity,
4. large size, and
5. presence of vascularity.^[18]^

In addition, and contrast-enhanced color Doppler ultrasonography could find the probability of malignancy with advanced accuracy, not only by enhancing the margin, depth, textural pattern of malignant soft tissue tumor on gray-scale US, and by visualizing vividly fine vascular structures developed in malignant tumors^[19]^; therefore, ultrasonography could be considered as a beneficial screening modality for malignant lipomatous neoplasm with non-invasiveness and cost-effectiveness. We suggest that sonographic examination should first be conducted with a screening modality for patients complaining of lipomatous tumors, and perform more accurate MRI examinations for patients with malignant features on sonographic examination.

After surgical removal of the lipomatous tumor, surgeons need to question the precision of the pathologic finding. In this case, a deep-seated, septate, intramuscular, and large-sized lipoma was pathologically confirmed as a benign lipoma after complete surgical excision in 2015. As reported in the previous literature, in the case of lipomatous tumors with this characteristic, the possibility of co-existence of both benign and malignant masses cannot be excluded.^[20]^ Although the possibility of co-existence, some histological sections with clustered and mosaic elements with and without cell atypia in the WDLS could mask the atypia and lead to a diagnosis of benign lipoma.

WDLSs can be classified as adipocytic, sclerosing, inflammatory, or spindle cells based on pathomorphology.^[21]^ In this case, the patient was diagnosed with adipocytic WDLS. Differential diagnosis of adipocytic WDLS from large benign lipoma occasionally presents similar histological characteristics, whereby the WDLS was identified predominantly with mature fat containing variable numbers of spindled cells displaying hyperchromatic nuclei and multivacuolated lipoblasts,^[22]^ immunohistochemistry is needed to perform accurate pathologic diagnosis of liposarcomas from benign lipomas. Currently, MDM2 and CDK4 using FISH immunochemical staining are considered as criterion standard methods for IHC.^[23–25]^ However, errors in interpretation of ICH have been reported in which benign lipomas with secondary changes with inflammatory cells stain positive with MDM2 ^[23]^ To diminish the challenges of histological diagnosis, P16 immunohistochemistry as an alternative marker combined with the MDM2-FISH immunochemical staining have recently been suggested as a diagnostic method to distinguish WDLS from deep-seated lipoma.^[24,26]^

As described above, in this patient, a large, deep-seated lipomatous mass, which was diagnosed as benign lipoma in 2015 and underwent surgical excision, recurred at the same site 4 years later as malignant liposarcoma with invasion to the ribs, pleural tissue, and surrounding muscles. In order to prevent recurrence that has progressed to malignancy, surgeons need to clarify the possibility of malignancy with appropriate imaging tests before surgery, to inform radiologists about the precise clinical impression that can lead to improved accuracy of assessment of malignant lipomatous tumors. If the radiological examination is unclear, in the case of lipomatous tumors showing a risk factor for recurrence before surgery, we suggest to perform surgical excision including an appropriate safety margin. For pathologic confirmation, if a malignant lipomatous tumor is strongly suspected before surgery, even if benign lipoma is confirmed by histological staining, we propose to perform immunohistochemistry considering the possibility of misdiagnosis on conditions agreed upon by the patient.

## 5 Conclusion

Through this case study, we suggest that deep-seated, septate, and giant lipomatous tumors should be considered as risk factors for recurrence with the possibility of malignancy and misdiagnosis. It is important to inform patients of all these possibilities and plan close and long-term follow-up.

To prevent recurrence, it is necessary to surgically confirm the safety margin for excision, to request a careful pathological examination of all histological sections based on the focality of nuclear atypia.

## Author contributions

**Data curation:** Yeon Ji Lee, Won Jin Cha, Yesol Kim.

**Supervision:** Deuk Young Oh.

**Validation:** Deuk Young Oh.

**Visualization:** Won Jin Cha, Yesol Kim.

**Writing – original draft:** Yeon Ji Lee.

**Writing – review & editing:** Deuk Young Oh.

## Abbreviations

Abbreviations: CDK4 = cyclin-dependent kinase 4, FISH = fluorescence in situ hybridization, IHC = immunohistochemistry, MRI = magnetic resonance imaging, WDLS = well-differentiated liposarcoma.
